# On the Core-Shell Nanoparticle in Fractional Dimensional Space

**DOI:** 10.3390/ma13102400

**Published:** 2020-05-22

**Authors:** A. Ali, M. A. Ashraf, Q. A. Minhas, Q. A. Naqvi, M. A. Baqir, P. K. Choudhury

**Affiliations:** 1 Department of Electronics, Quaid-i-Azam University, Islamabad 45320, Pakistan; aijazali@ele.qau.edu.pk (A.A.); aqueel@qau.edu.pk (M.A.A.); qminhas@qau.edu.pk (Q.A.M.); qaisar@qau.edu.pk (Q.A.N.); 2 Department of Electrical and Computer Engineering, Sahiwal Campus, COMSATS University Islamabad, Islamabad 57000, Pakistan; abuzargardazi@yahoo.com; 3 Institute of Microengineering and Nanoelectronics, Universiti Kebangsaan Malaysia, UKM Bangi, Selangor 43600, Malaysia

**Keywords:** core-shell nanoparticle, surface plasmon, fractional dimension space, polarizability, scattering

## Abstract

The investigation of core-shell nanoparticles has been greatly exciting in biomedical applications, as this remains of prime importance in targeted drug delivery, sensing, etc. In the present work, the polarizability and scattering features of nanoparticles comprised of nano-sized dielectric/metallic core-shell structures were investigated in the fractional dimensional (FD) space, which essentially relates to the confinement of charged particles. For this purpose, three different kinds of metals—namely aluminum, gold and silver—were considered to form the shell, having a common silicon dioxide (SiO_2_) nanoparticle as the core. It is noteworthy that the use of noble metal-SiO_2_ mediums interface remains ideal to realize surface plasmon resonance. The core-shell nanoparticles were considered to have dimensions smaller than the operating wavelength. Under such conditions, the analyses of polarizability and the scattering and absorption cross-sections, and also, the extinction coefficients were taken up under Rayleigh scattering mechanism, emphasizing the effects of a varying FD parameter. Apart from these, the tuning of resonance peaks and the magnitude of surface plasmons due to FD space parameter were also analyzed. It was found that the increase of FD space parameter generally results in blue-shifts in the resonance peaks. Apart from this, the usage of gold and silver shells brings in fairly large shifts in the peak positions of wavelengths, which allows them to be more suitable for a biosensing purpose.

## 1. Introduction

The core-shell forms of nanoparticles have been in the research limelight in the context of plasmonic-based studies [1]. More importantly, these find exciting applications in the biomedical area of research as the study includes targeted drug delivery, biosensing, bioimaging, targeted gene delivery, DNA interactions, pH nano-sensor for single cell, etc. [2,3,4,5,6,7]. In this stream, possible control over the plasmonic parameters led to the interest of the research and development community to explore varieties of electromagnetic features [8,9] of core-shell nanoparticles in different ambiance. The current advancements in nanotechnology envisions the barely perceptible phenomena produced by either single nanoparticles [10] or its collective response [11].

In the context of plasmons, the commonly studied noble metals are silver (Ag) and gold (Au), as these exhibit strong surface plasmon resonance (SPR) over a wide wavelength span [12,13,14,15,16,17,18,19]—a phenomenon that may be modified by tailoring the structure, shape and size of the nanoparticle [20]. Reference [21] demonstrates that the use of SiO_2_ provides stability in core-shell nanoparticles. However, different types of core-shell nanoparticles have been used to enhance the performance of photovoltaic solar cell and improve the photocatalytic activity [22,23,24].

The most canonical problems facilitating the analyses of surface plasmon exploit the scattering of electromagnetic waves by a spherical nanoparticle [8]. Within the context, fractional calculus deals with the situations wherein the order of the operator can be arbitrarily complex-valued [25,26]. In this stream, the fractional dimensional (FD) space remains a key tool to analyze problems in physics and engineering [27,28,29]. While simple geometries and smooth surfaces can be represented using canonical shapes, most of the practical objects embody irregularity and complexities in shape, and thus, are modeled using the FD space [30].

In recent years, researchers have generalized the integer dimensional space for the case of FD space exploiting the concept of fractional calculus, in order to describe the fractional solutions of certain electromagnetic problems [31,32]. Reference [33] describes the formalism for integration in the FD space. In reference [34] the solutions for Laplace and Poisson equations for potentials in the FD space have been analyzed. The general plane wave and cylindrical wave solutions to the wave equations in the FD space have been reported in [35]. An anisotropic medium is treated as an isotropic FD space whose dimension is determined by the degree of anisotropy [36].

In the present work, we aim at investigating the interaction of light with the core-shell metallodielectric nanoparticles embedded in the FD space, which determines the confinement of charged particles. We take three different types of metallic shells, viz. aluminum (Al), gold (Au), and silver (Ag). To the best of the authors’ knowledge, this kind of electromagnetic interaction has not yet been reported in the literature. To be more precise, we touch upon the studies of polarizability and scattering, absorption, and extinction coefficients for the core-shell metallodielectric nanoparticles in the FD space with special emphasis on the effect of FD space parameter and nanoparticle dimension on these electromagnetic characteristics. A greater FD space parameter indicates less confinement of charged particles. It has been found that the localized plasmonic behavior leaves significant impact on the response in respect of the stated interaction features, and can be tuned by suitably using the FD space parameter. Additionally, the resonance can be tuned by altering the core/shell radii of the nanoparticle. As such, the desired resonance conditions can be attained by suitably tailoring the parametric values used for the constituents of nanoparticle. As the core-shell forms of nanoparticles have been proved to be prudent for biomedical applications, the present study would be useful in further developments in the relevant research area.

## 2. Model and Theory

**Figure 1:**
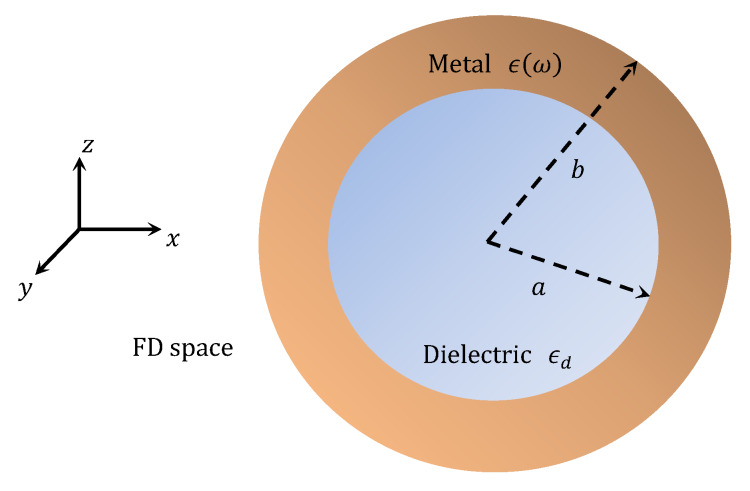
Schematic of the spherical shaped core-shell nanoparticle in the FD space.

Figure 1 shows the schematic of metallodielectric core-shell nanoparticle in the FD space. We consider a z-polarized plane wave, propagating along the x-direction, excites the nanoparticle. As such, the expression for incidence electric field (${\bar{E}}_{inc}$) associated the with the plane wave can be written as

(1) $${\bar{E}}_{inc}=\hat{z}E_{0}e^{-ikx},$$

where k ($=\omega \surd \left(\mu_{0}\epsilon_{0}\right)$) and $E_{0}$ are the wavenumber and amplitude of the incident field, respectively. Additionally, ω is the frequency of monochromatic wave in the optical regime, and $\mu_{0}$ and $\epsilon_{0}$ are the free-space permeability and permittivity, respectively. We suppress the time t-harmonic factor ($e^{-i\omega t}$) throughout the analytical discussion.

In the context of scattering, the Mie scattering theory is used to study the scattering from an object with its size comparable to (or larger than) the operating wavelength. The use of Rayleigh scattering approximation remains valid if the particle size is smaller than the operating wavelength. In the present work, as the size of the nanoparticles is very small when compared to the wavelength of incidence light (i.e., b≪λ), the quasi-static approximation can be used. Accordingly, Equation (1) can be approximated as

(2) $${\bar{E}}_{inc}=\hat{z}E_{0}.$$

At this point, it must be stated that, as the particle size is much smaller than the operating wavelength of the oscillating electric field (of the incident light), it induces an oscillating dipole moment in the particle which, in turn, radiates light in the pattern of dipole. As such, the situation appears to be as if a sphere is embedded in a uniform static electric field. This suggests an existing connection between the electrostatic fields and scattering by the particle (having a small dimension as compared to the operating wavelength). Therefore, one has to solve the Laplace equation (i.e., $\nabla^{2}\varphi =0$; φ being the electrostatic potential) supporting the light-matter interaction [37]. Further, the space represents an effective physical description of confinement in low-dimensional systems [36]. Within the context, Muslih et al. [33] firstly introduced the concept of fractional space to solve the electrostatic problem. This triggers the concept of FD space to be exploited while solving the Laplace equation in connection with the scattering of light by subwavelength-sized nanoparticles.

The incident plane wave ${\bar{E}}_{\left(f,inc\right)}$ under the quasi-static approximation in the FD space can be described as [34]

(3) $${\bar{E}}_{\left(f,inc\right)}=\hat{z}\left(\alpha -2\right)E_{0},$$

where α (such that 2<α<3) is the FD space parameter. It is noteworthy that the situation corresponding to α=3 reduces the FD space to the ordinary three-dimensional (3D) space [34,36]. It is worth mentioning at this point that the fractional value of α agrees with the experimental physical observations [38], and also, the discrepancy between the theoretical and experimental values of the anomalous magnetic moment of the electron could be resolved [39].

In the FD space, the potentials in the core ($\varphi_{c}$), shell ($\varphi_{s}$) of the nanoparticle and the surrounding medium ($\varphi_{b}$) are obtained to be [40,41,42]

(4) φc=−E0α2(α−2)ϵsϵb{ϵs+(α−1)ϵb}{ϵc+(α−1)ϵs}+(ab)α(α−1)(ϵs−ϵb)(ϵc−ϵs)rcosθ,

(5) φs=−E0[α{ϵc+(α−1)ϵs}−α(ϵc−ϵs)(ab)α](α−2)ϵb{ϵs+(α−1)ϵb}{ϵc+(α−1)ϵs}+(ab)α(α−1)(ϵs−ϵb)(ϵc−ϵs)rcosθ,

(6) φb=−E0(α−1)rcosθ+E0cosθ[(ϵs−ϵb){ϵc+(α−1)ϵs}+(ab)α(ϵc−ϵs){ϵb+(α−1)ϵs}]bα{ϵs+(α−1)ϵb}{ϵc+(α−1)ϵs}+(ab)α(α−1)(ϵs−ϵb)(ϵc−ϵs)r2.

In these equations, $\epsilon_{c}$, $\epsilon_{s}$, and $\epsilon_{b}$ are the permittivity values of core, shell (of nanoparticle) and the surrounding medium, respectively. As stated before, the choice of α=3 reduces Equations (4)–(6) to the corresponding ordinary 3D space [40] forms.

When a uniform electromagnetic field impinges a nanoparticle, it generates the dipole moment on the surface of it. The potential $\varphi_{dip}$ for an ideal dipole at a distance r in an unbounded medium can be written as [43]

(7) $$\varphi_{dip}=\frac{p\cdot r}{4\pi \varepsilon_{b}r^{3}}=\frac{p\cos\theta }{4\pi \varepsilon_{b}r^{3}},$$

where p is the dipole moment, which, due to the electric polarizability of the core-shell nanoparticle, can be written as [43]

(8) p=4πεbbαE0[(εs−εb){ϵc+(α−1)ϵs}+(ab)α(εc−εs){ϵb+(α−1)ϵs}][{ϵs+(α−1)ϵb}{ϵc+(α−1)ϵs}+(ab)α(α−1)(εc−εb)(εb−εs)].

As such, the potential $\varphi_{dip}$ due to dipole can be written as

(9) φdip=E0[(εs−εb){ϵc+(α−1)ϵs}+(ab)α(εc−εs){ϵb+(α−1)ϵs}]bα[{ϵs+(α−1)ϵb}{ϵc+(α−1)ϵs}+(ab)α(α−1)(εc−εb)(εb−εs)]r2cosθ.

The applied field induces a dipole moment p. The ease with which the sphere is polarized, may be specified by the polarizability $P_{0}$, which is related to p as

(10) $$p=\varepsilon_{b}P_{0}E_{0}.$$

The dipole term present in the expression for potential $\varphi_{b}$ corresponding to the surrounding medium, provides the polarizability $P_{0}$ of the nanoparticle, which can be written as [41]

(11) P0=4πr3[3(ϵs−ϵb){ϵc+(α−1)ϵs}+(ab)α(ϵc−ϵs){ϵb+(α−1)ϵs}]{ϵs+(α−1)ϵb}{ϵc+(α−1)ϵs}+(ab)α(α−1)(ϵs−ϵb)(ϵc−ϵs).

Now, the absorption and scattering cross-sections of nanoparticle can be obtained by exploiting the expression for polarizability in Equation (8) [41]. Within the context, the scattering cross-section $\sigma_{sca}$ is directly related to the squared magnitude of polarizability, and therefore, it may be expressed as

(12) $$\sigma_{sca}=\frac{1}{6\pi \epsilon_{b}^{2}}k^{4}{\left|P_{0}\right|}^{2}.$$

Additionally, the absorption cross-section $\sigma_{abs}$ is proportional to the imaginary part (Im) of polarizability, and is expressed as

(13) $$\sigma_{abs}=\frac{k}{\epsilon_{b}}Im\left(P_{0}\right).$$

The extinction coefficient $\sigma_{ext}$ may be obtained by considering the summation of scattering and absorption cross-sections.

Now, the wavelength-dependent permittivity of metal can be deduced by exploiting the Lorentz-Drude (LD) model. Mathematically, it can be described as [44]

(14) $$\varepsilon \left(\omega \right)=1-\frac{f_{0}\omega_{p}^{2}}{\omega \left(\omega -i\gamma_{0}\right)}+\overset{m}{\underset{j=1}{\sum }}\frac{f_{j}\omega_{p}^{2}}{\left(\omega_{j}^{2}-\omega^{2}\right)+i\omega \gamma_{j}},$$

where $\omega_{p}$ is the plasma frequency of bulk material, m is the number of oscillators and $f_{0}$ is the resonant frequency of individual oscillator. The number of oscillators m is taken as m=5. Additionally, $f_{j}$ is the oscillator strength and $\gamma_{j}$ is the decaying constant for the $j^{th}$ oscillator.

**Figure 2:**
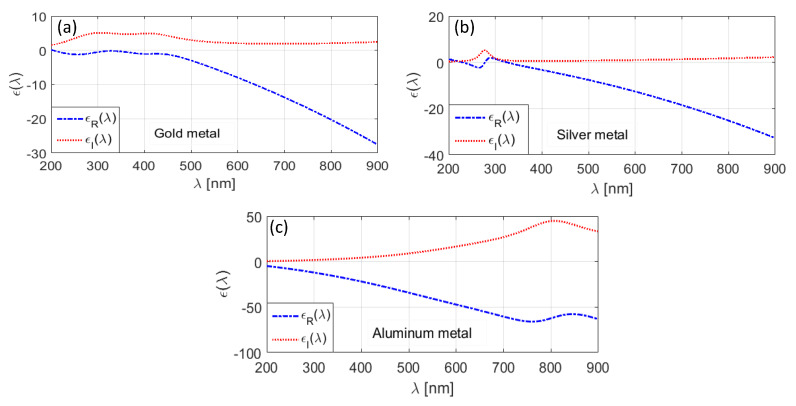
Wavelength-dependence of permittivity for (**a**) Au, (**b**) Ag, and (**c**) Al.

Figure 2a–c, respectively, illustrates the wavelength-dependence of dielectric permittivity corresponding to three different metals, namely gold (Au), silver (Ag), and aluminum (Al) in the wavelength span of 200 to 900 nm. These have been deduced by exploiting the LD model, using the parametric values, as used in Reference [44] for the computational purpose. In all these figures, the real $\epsilon_{R}\left(\lambda \right)$ and the imaginary $\epsilon_{I}\left(\lambda \right)$ parts of permittivity are plotted against wavelength λ. We observe that $\epsilon_{R}\left(\lambda \right)$ remains negative in the wavelength range of operation, and also, the increase in wavelength makes it more negative. However, the imaginary parts $\epsilon_{I}\left(\lambda \right)$ for all the three types of metals are positive.

Figure 2 shows that, among the chosen metals, Al exhibits more negative $\epsilon_{I}$ permittivity component and more positive $\epsilon_{R}$ permittivity component with the increase in wavelength (Figure 2c). Within the context, it is worth to mention that the positive value of permittivity results in losses, whereas the negative value of the same remains essential to achieve the surface plasmon (SP) waves. We observe in Figure 2 that Al exhibits the maximum loss among the chosen metals. For the other two types of metals, the amount of loss is very small, based on the wavelength dependence of permittivity values. Using the stated three different types of metals as the shell, encapsulating the nano-sized SiO_2_ dielectric medium, would be interesting to study as the structure forms nano-structured metal-dielectric interface, thereby generating the SP waves.

## 3. Results and Discussion

**Figure 3:**
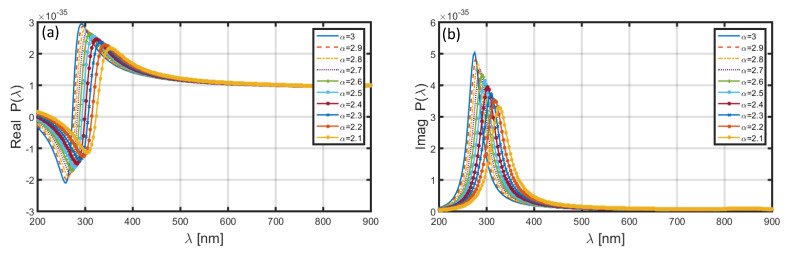
Wavelength-dependence of the (**a**) real and (**b**) imaginary parts of polarizability of the nanoparticle coated with Al.

In the very first attempt, we evaluate the polarizability of nanoparticle in the 400 to 900 nm wavelength span. Figure 3 illustrates the case when we consider the nanoparticle as having Al shell (and SiO_2_ core); the real and imaginary parts of polarizability are plotted in Figure 3a,b, respectively, corresponding to different values of FD space parameter α, namely 2.1, 2.2, 2.3, 2.4, 2.5, 2.6, 2.7, 2.8, 2.9, and 3.0. However, as stated before, using α=3.0 makes the space to be of the ordinary 3D kind. Furthermore, in order to plot these, we consider the values of radius of the core (i.e., a) and shell (i.e., b) to be 1.6 and 2.0 nm, respectively. As such, the thickness of Al nanolayer remains to be 0.4 nm.

Following Equation (11), it is obvious that the polarizability depends on the FD space parameter, and therefore α essentially alters the dielectric property of nanoparticles. As such, different values of α alter the distribution of charged ions in the FD space, thereby affecting the resonance conditions.

We observe in Figure 3a that the choice of α=2.1 yields the resonance peak to be in the vicinity of λ~350 nm, and also, the increase in the value of α results in blue-shifts in the position of peak. Corresponding to α=3.0, the resonance peaks remains at λ~290 nm. The presence of resonance peaks is attributed to the phenomenon of localized surface plasmon resonance (LSPR). As to the imaginary part of polarizability (Figure 3b), in this case as well, the positions of resonance peaks exhibit blue-shifts with the increase in α; corresponding to α=2.1, the peak is positioned at λ~330 nm. We also notice that, in Figure 3a,b the peak polarizability of nanoparticle increases with increasing α.

**Figure 4:**
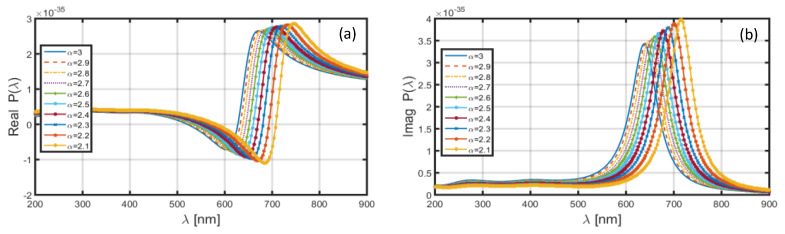
Wavelength-dependence of the (**a**) real and (**b**) imaginary parts of polarizability of the nanoparticle coated with Au.

Upon replacing the Al shell by Au shell in the nanoparticle, and keeping the core-shell dimensional features similar to that used above (in the case of Al shell), the achieved wavelength dependence of polarizability is illustrated in Figure 4. We observe in this case that, corresponding to both the real (Figure 4a) and imaginary (Figure 4b) parts of polarizability, the positions of resonance peaks are significantly altered to the higher wavelengths. However, Figure 4a,b exhibits blue-shifts in the resonance peaks with the increase in FD space parameter α, and also, the peak polarizability decreases with the increase in α. To be more explicit, Figure 4a shows the position of resonance peak corresponding to α=2.1 as near λ~750 nm; for α=3.0, it shifts to λ~670 nm. Similarly, in Figure 4b, the choice of α=2.1 results in the resonance peak to be at λ~720 nm, which moves to λ~640 nm when the value of α is increased to 3.0. As such, the increase in α from 2.1 to 3.0 causes a blue-shift of ~80 nm in the resonance peak for both the real and imaginary parts of polarizability.

**Figure 5:**
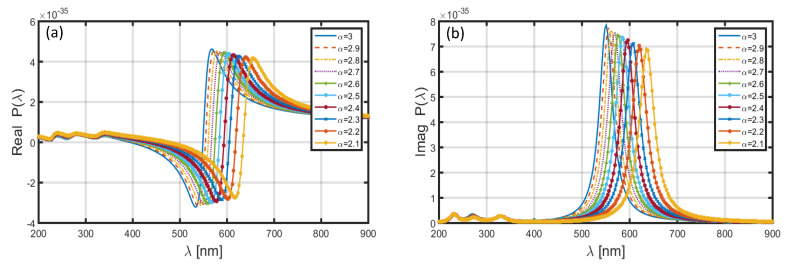
Wavelength-dependence of the (**a**) real and (**b**) imaginary parts of polarizability of the nanoparticle coated with Ag.

The use of Ag shell in the nanoparticle (of the similar dimensional characteristic as above) yields the wavelength-dependence of polarizability, as depicted in Figure 5. Though the spectral features of the achieved polarizability in this figure have similar trend as observed in Figure 3 and Figure 4, the λ-positions of resonance peaks corresponding to different values of FD parameter lie in between those achieved corresponding to the cases of Al (Figure 3) and Au (Figure 4) shells. The common characteristic remains in all the three situations that the increase in α results in blue-shifts in the resonance peaks. However, in the cases of Al and Ag shells in the nanoparticle, the peak magnitudes of the real and imaginary parts of polarizability increase (though Al shows more increase as compared to Ag) with the increase in α, whereas they exhibit decrease (with increasing α) in the case of Au shell.

We now look at the other electromagnetic interaction characteristics that include the wavelength dependence of scattering ($\sigma_{sca}$) and absorption ($\sigma_{abs}$) cross-sections, and also, the extinction coefficients ($\sigma_{ext}$) in the 200 to 900 nm wavelength span. For this purpose, we consider two different sets of dimensional features of the aforementioned types of core-shell nanoparticle, viz. as a=1.6 nm, b=2 nm and a=60 nm, b=80 nm, under the conditions of the FD parametric values used above, i.e., 2.1, 2.2, 2.3, 2.4, 2.5, 2.6, 2.7, 2.8, 2.9, and 3.0.

**Figure 6:**
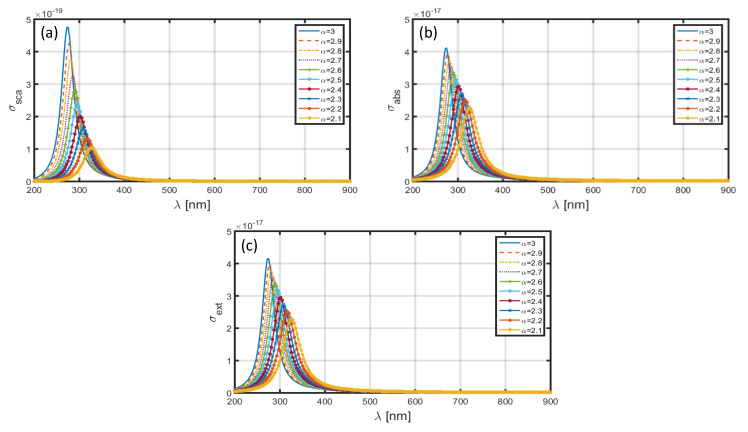
Plots of (**a**) scattering cross-section, (**b**) absorption cross-section, and (**c**) extinction coefficient against λ for SiO_2_-Al nanoparticle in the FD space corresponding to the dimensions a=1.6 nm, b=2 nm.

Figure 6 depicts the $\sigma_{sca}$–λ (Figure 6a), $\sigma_{abs}$–λ (Figure 6b) and $\sigma_{ext}$–λ (Figure 6c) plots for different α-values in the case when Al is used as the nanoparticle shell; the core section is comprised of SiO_2_ dielectric medium. As stated before, we use the dimensional features as a=1.6 nm and b=2 nm (Figure 1). In Figure 6a, we observe that the scattering cross-section exhibits the highest value corresponding to α=3, i.e., when the FD space is reduced to the ordinary 3D space. The use of α=3 exhibits the resonance peak to be at λ∼275 nm; the decrease in α results in red-shifts in the positions of resonance peaks, and also, the scattering cross-section decreases significantly (with decreasing α). A similar trend can be observed corresponding to $\sigma_{abs}$ as well as $\sigma_{ext}$ in Figure 6b,c, respectively. However, these differ in terms of the magnitudes (with changing α) of resonance peaks, which is larger corresponding to the scattering cross-section than the absorption cross-section; the extinction coefficient is essentially governed by the scattering and absorption cross-sections. To be more specific, Figure 6a,b determine the red-shifts in the resonance peaks for $\sigma_{sca}$ and $\sigma_{abs}$ to be ~60 and ~50 nm, respectively, with the decrease in the value of α from 3.0 to 2.1.

**Figure 7:**
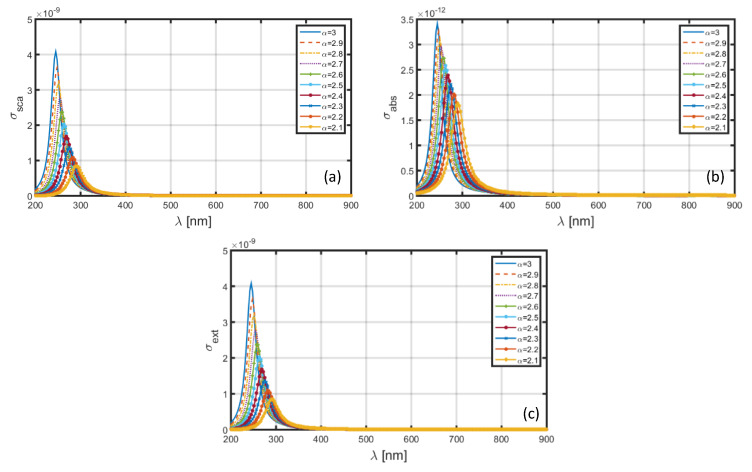
Plots of (**a**) scattering cross-section, (**b**) absorption cross-section, and (**c**) extinction coefficient against λ for SiO_2_-Al nanoparticle in the FD space corresponding to the dimensions a=60 nm, b=80 nm.

The dimensional features of nanoparticles remain responsible for the electromagnetic characteristics. With this viewpoint in mind, we can now significantly increase the dimensions of core as well as shell to the values represented by a=60 nm and b=80 nm (Figure 1); Figure 7 exhibits the obtained wavelength-dependence patterns of $\sigma_{sca}$ (Figure 7a), $\sigma_{abs}$ (Figure 7b), and $\sigma_{ext}$ (Figure 7c). We observe in these figures that, although the factors under investigation exhibit red-shifts in the resonance peaks having the patterns as seen in Figure 6, the magnitudes (of resonance peaks) are now significantly increased. This is very much expected owing to the nanoparticle dimension being large enough to yield larger scattering of waves. Simultaneously, the absorption cross-section and extinction coefficients also increase. Figure 7 shows that the value of red-shift is ~50 nm (as α is increased from 2.1 to 3.0) for both $\sigma_{sca}$ and $\sigma_{abs}$, which is less than that noticed in the case of Al shell (Figure 6). We also observe in Figure 7 that the increase in dimension generally results in blue-shifts in the resonance peaks, as compared with that observed in Figure 6 (with low core-shell dimensional values), obtained for different α-values. As such, the electromagnetic characteristics of nanoparticle will essentially depend on the LSPR conditions which, in turn, depend on the dimensional features of core-shell nanoparticle and the FD space conditions.

**Figure 8:**
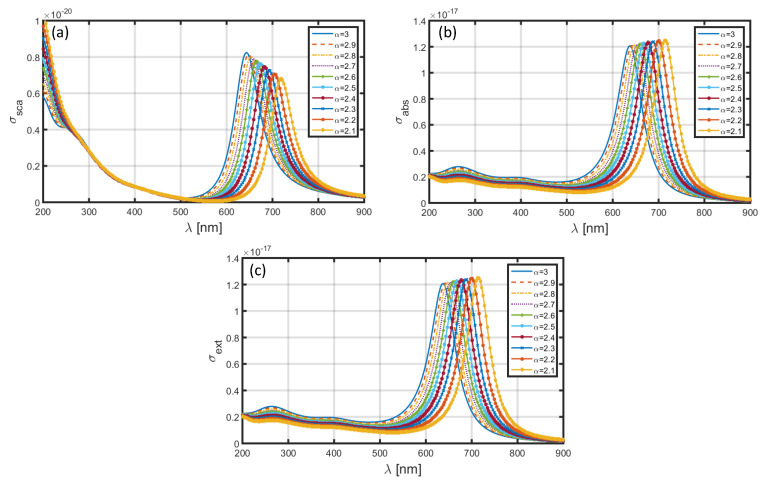
Plots of (**a**) scattering cross-section, (**b**) absorption cross-section, and (**c**) extinction coefficient against λ for SiO_2_-Au nanoparticle in the FD space corresponding to the dimensions a=1.6 nm, b=2 nm.

**Figure 9:**
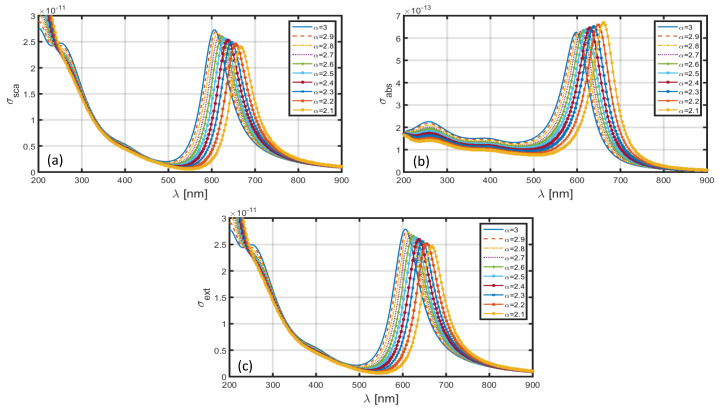
Plots of (**a**) scattering cross-section, (**b**) absorption cross-section, and (**c**) extinction coefficient against λ for SiO_2_-Au nanoparticle in the FD space corresponding to the dimensions a=60 nm, b=80 nm.

Figure 8 and Figure 9 illustrate the wavelength-dependence of $\sigma_{sca}$, $\sigma_{abs}$ and $\sigma_{ext}$ corresponding to the sets of dimensional features of core-shell nanoparticle as a=1.6 nm, b=2 nm and a=60 nm, b=80 nm, respectively, when the nanoparticle has an Au shell; the core section is comprised of the same medium, i.e., SiO_2_. We make changes in the FD space parameter in the similar form, as previously done. We observe that the choice of Au shell results in significant amount of red-shift in the positions of resonance peaks, as compared to those in Figure 6 and Figure 7 (with the usage of Al as shell). More precisely, in the case of a=1.6 nm, b=2 nm, the peak positions corresponding to $\sigma_{sca}$ (Figure 8a), $\sigma_{abs}$ (Figure 8b) and $\sigma_{ext}$ (Figure 8c) lie in the wavelength range ~640−720 nm, with the decreasing values of α resulting into red-shifts of ~80 nm in the resonance peaks. The magnitudes of factors under investigations are only a little affected with changing FD parameter. Another noticeable fact remains that, with changing values of α, the obtained peak positions are more resolved with less difference in magnitudes in this case, as compared to that observed in Figure 6. The increase in nanoparticle dimension to a=60 nm, b=80 nm causes two-fold, viz. the resonance peaks exhibit significant blue-shifts of about 45 nm, as compared to what we see in Figure 8, and also, the magnitudes of $\sigma_{sca}$ (Figure 9a), $\sigma_{abs}$ (Figure 9b), and $\sigma_{ext}$ (Figure 9c) exhibit a marked increase. This is as physically expected, owing to the usage of large nanoparticle dimension in this case.

**Figure 10:**
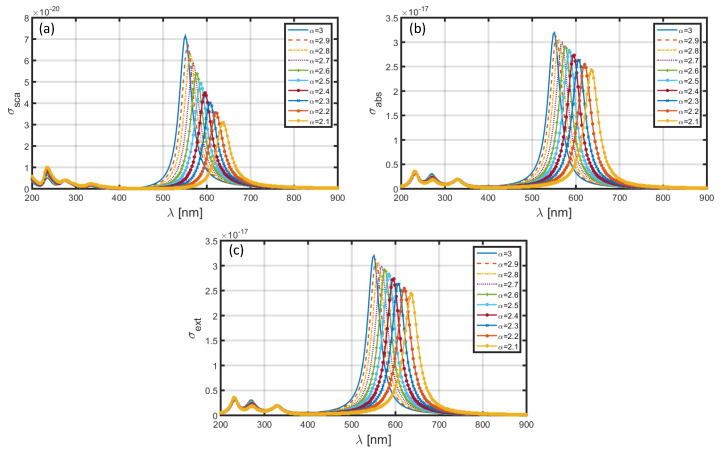
Plots of (**a**) scattering cross-section, (**b**) absorption cross-section, and (**c**) extinction coefficient against λ for SiO_2_-Ag nanoparticle in the FD space corresponding to the dimensions a=1.6 nm, b=2 nm.

**Figure 11:**
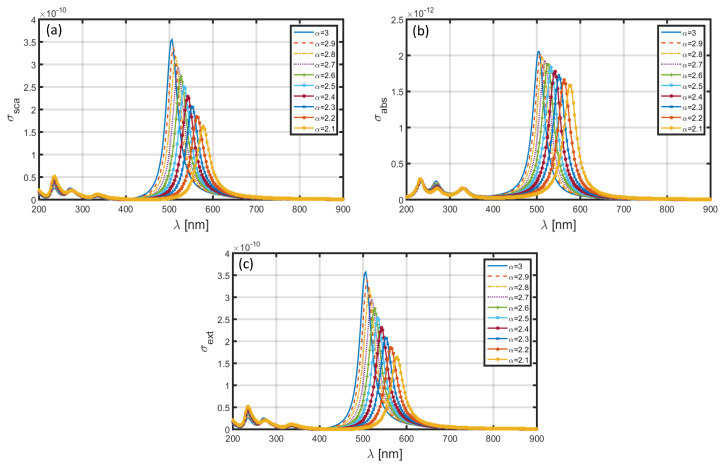
Plots of (**a**) scattering cross-section, (**b**) absorption cross-section, and (**c**) extinction coefficient against λ for SiO_2_-Ag nanoparticle in the FD space corresponding to the dimensions a=60 nm, b=80 nm.

Upon using Ag shell in the nanoparticle, and keeping the core as comprised of SiO_2_ dielectric medium, the obtained $\sigma_{sca}$–λ, $\sigma_{abs}$–λ and $\sigma_{ext}$–λ plots corresponding to different α-values (as used before) are shown in Figure 10 and Figure 11. These figures, respectively, illustrate the cases of core-shell nanoparticle dimensions as a=1.6 nm, b=2 nm, and a=60 nm, b=80 nm. The observed spectral features make it explicit that the magnitudes of resonance peaks corresponding to $\sigma_{sca}$, $\sigma_{abs}$ and $\sigma_{ext}$ significantly alter with the changing values of the FD parameter—a feature in contrast to the situation when the Au shell was in use (Figure 8 and Figure 9) where the magnitudes of resonance peaks were less different in values with changing values of α. Furthermore, the positions of these resonance peaks lie in the range ~550–630 nm in Figure 10 and ~500–580 nm in Figure 11. As such, the usage of Ag shell yields red-shifts of ~275 nm in the peak position as compared to the case when the Al shell is used (Figure 6 and Figure 7), and exhibits blue-shifts of ~90 nm as compared to situation of Au shell in use (Figure 8 and Figure 9). In this case as well, we observe significant increase in $\sigma_{sca}$, $\sigma_{abs}$, and $\sigma_{ext}$ upon using the core-shell nanoparticle of large dimension. Apart from these, the other electromagnetic features exhibit almost similar trends noticed in the cases of nanoparticles having Al and/or Au shells.

Upon using Ag shell in the nanoparticle, and keeping the core as comprised of SiO_2_ dielectric medium, the obtained $\sigma_{sca}$-λ, $\sigma_{abs}$-λ and $\sigma_{ext}$-λ plots corresponding to different α-values (similar to as used before) are shown in Figure 10 and Figure 11. These figures, respectively, illustrate the cases of core-shell nanoparticle dimensions as a=1.6 nm, b=2 nm, and a=60 nm, b=80 nm. The observed features make it explicit that the magnitudes of resonance peaks corresponding to $\sigma_{sca}$, $\sigma_{abs}$ and $\sigma_{ext}$ significantly alter with changing values of FD space parameter—a feature in contrast to the situation when Au shell was in use (Figure 8 and Figure 9) where the magnitudes were less different in values with changing α. Furthermore, the positions of these peaks lie in the range ~550–640 nm in Figure 10 and ~505–580 nm in Figure 11. As such, the usage of Ag shell yields red-shifts in the peak position as compared to the case when Al shell is used (Figure 6 and Figure 7), and exhibits blue-shifts as compared to situation of Au shell in use (Figure 8 and Figure 9). In this case as well, we observe significant increase in $\sigma_{sca}$, $\sigma_{abs}$ and $\sigma_{ext}$ upon using the core-shell nanoparticle of large dimension. Apart from these, the other electromagnetic features exhibit almost similar trends noticed in the cases of nanoparticles having Al and/or Au shells. For instance, blue-shifts (of nearly 75 nm) in the resonance peaks happen with increasing the FD space parameter from 2.1 to 3.0.

Table 1 summarizes the results obtained above, corresponding to the cases of core-shell dimensions with the radii a, b as 1.6 nm, 2.0 nm, and 60 nm, 80 nm, corresponding to certain values of FD space parameter α, namely 2.1, 2.5 and 3.0. We particularly emphasize on the scattering and absorption cross-sections, and also, the extinction coefficients, as observed in Figure 6, Figure 7, Figure 8, Figure 9, Figure 10 and Figure 11. From this table, shifts in resonance peaks can be estimated while different forms of metallic shells are used in the formation of nanoparticles.

The aforesaid discussion indicates that the resonance peaks corresponding to the scattering and absorption cross-sections can be tuned by suitably tailoring the parametric values of the core-shell nanoparticles and the operating conditions. Such adjustments essentially alter the LSPR condition, which causes shifts in the positions of resonance peaks. Furthermore, the increase in FD space parameter introduces blue-shifts in resonance peaks, which assume large values when the Au and Ag shell are used in the nanoparticle. It is to be added at this point that the obtained results are derived upon exploiting the quasi-static approximation, which implements the theory of Rayleigh scattering as the size of nanoparticle is very small as compared to the wavelength of incidence light. The theory can be extended to the case of spherical/cylindrical Bessel functions in the FD space as well, which essentially will require a new approach to deduce the polarizability and scattering cross-section—work that will be taken-up in a future communication. As the core-shell nanoparticles have been promising for varieties of biomedical applications, the present communication would be useful in further studies in support of designing such nanoparticles for on-demand kinds of sensing needs.

## 4. Conclusions

Considering the core-shell kind of nanoparticles of different geometrical parameters, light-matter interaction was investigated in the FD space by employing the Rayleigh mechanism. This is because the operating wavelength span was considerably large as compared to the dimensions of nanoparticles under investigation. Three different types of metallic shells were taken into account—namely Al, Au and Ag. The wavelength-dependent permittivity values of metallic shells were deduced by employing the LD model. Further, the wavelength-dependent tuning of resonance peaks was noticed with respect to the variations in FD parameter and the core-shell radii of nanoparticles. It was noticed that the FD space holding the nanoparticle leaves significant impact on the resonance properties. The increase in FD parameter generally causes blue-shifts in resonance peaks. These shifts are relatively large while the Au and Age shells are used in the nanoparticle. It is noteworthy that a higher FD parameter indicates lesser confinement of charged particles in space. Additionally, the magnitude of resonance peaks gets largely affected due to varying dimension of nanoparticle undergoing interaction with light. It is expected that such studies would be useful in sensing applications in the biomedical arena.

## Figures and Tables

**Table 1:** Resonance peak positions as observed corresponding to the scattering and absorption cross-sections, and also, the extinction coefficients (as discussed in Figure 6, Figure 7, Figure 8, Figure 9, Figure 10 and Figure 11).

Shell Material	FD Parameter α	Peak Positions of Wavelength (nm)					
		a=1.6 nm; b=2.0 nm			a=60 nm; b=80 nm		
		$\sigma_{abs}$	$\sigma_{sca}$	$\sigma_{ext}$	$\sigma_{abs}$	$\sigma_{sca}$	$\sigma_{ext}$
Al	2.1	326	326	326	290	290	290
	2.5	296	296	296	263	263	263
	3.0	275	275	275	245	245	245
Au	2.1	716	719	719–	665	668	670
	2.5	668	674	674	626	629	629
	3.0	638	644	644	605	599	599
Ag	2.1	635	638	638	578	578	578
	2.5	584	587	587	533	533	533
	3.0	551	551	551	503	502	506
